# PREDICTING CARE-RECIPIENTS’ WELLBEING BASED ON SPOUSAL CAREGIVERS’ CO-OCCURRING STRESSORS AND RESOURCES

**DOI:** 10.1093/geroni/igac059.3112

**Published:** 2022-12-20

**Authors:** Ruotong Liu, Iris Chi, Shinyi Wu

**Affiliations:** Oregon Health & Science University, Marina del Rey, California, United States; University of Southern California, Los Angeles, California, United States; University of Southern California, Los Angeles, California, United States

## Abstract

Besides caregivers, care-recipients’ health outcomes can be impacted by caregiving as well. The aim of the current study is to explore whether care-recipients’ longitudinal health outcomes differ by caregivers’ profiles characterized by the co-occurring and relative intensities of caregiving stressors and resources. Data from Round 5 and Round 7 of National Study of Caregiving and Round 5 to Round 8 of the linked National Health and Aging Trends Study are utilized. After multiple imputation, sample characteristics are described and compared among the 639 unique care-recipients by caregivers’ latent classes. Generalized estimating equations (GEE) with first-order autoregressive covariance are estimated to examine the differences in care recipients’ health outcomes at baseline and rates of change across groups. The average self-rated health at baseline is 2.73 (SD=1.02), and the average baseline depressive symptoms are 2.65 (SD=2.76). GEE results indicate that compared to care-recipients cared by low-stress low-support spousal caregivers, those cared by medium-stress high-support and high-stress medium-support partners score 0.20 and 0.35 unit lower in self-rated health at baseline, respectively, and those cared by high-stress medium-support caregivers on average score 1.21 unit higher in depressive symptoms at baseline. No statistical differences in rate of change are detected for both self-rated health and depressive symptoms across groups. Despite different levels of health at baseline, our research does not find care-recipients’ rates of change in health different across groups over one-year. Future studies are needed to further explore the longer-term differences in rates of change and to better understand the caregiving dyads.

